# Antibacterial and Antibiofilm Activities of Hydralazine, an Antihypertensive Drug: In Vitro and In Silico Approaches

**DOI:** 10.3390/antibiotics14030286

**Published:** 2025-03-10

**Authors:** Antônio Mateus Gomes Pereira, Benise Ferreira da Silva, Ingrid Maria Frota Araujo, Francisco Kauê Carvalho Aguiar, Paulo Adenes Teixeira Coelho, Renata Albuquerque Costa, Marcia Machado Marinho, Emmanuel Silva Marinho, João Victor Serra Nunes, Victor Alves Carneiro, Hélcio Silva dos Santos

**Affiliations:** 1 Graduate Program in Biotechnology, Northeast Network of Biotechnology (RENORBIO), State University of Ceará, Campus Itaperi, Fortaleza 60714-903, Brazil; mathewsgomes20@gmail.com (A.M.G.P.); benise.f.silva@gmail.com (B.F.d.S.); 2 Center of Bioprospection and Experimentation Molecular Applied (NUBEM), University Center INTA–UNINTA, Sobral 62050-100, Brazil; frotaingrid730@gmail.com (I.M.F.A.); kcaguiar09@gmail.com (F.K.C.A.); pauloadenes11@gmail.com (P.A.T.C.); renata.albuq@gmail.com (R.A.C.); victor.carneiro@uninta.edu.br (V.A.C.); 3 Center for Exact Sciences and Technology, Vale do Acaraú University, Sobral 62040-370, Brazil; marinho.marcia@gmail.com; 4 Postgraduate Program in Natural Sciences, Ceará State University, Fortaleza 60714-903, Brazil; emmanuel.marinho@uece.br; 5 Analytical Centre, Department of Physics, Federal University of Ceará, Fortaleza 60020-181, Brazil; jvictorsn@centralanalitica.ufc.br

**Keywords:** secondary use, hydralazine, antibacterial, antibiofilm

## Abstract

**Background:** The rise of multidrug-resistant (MDR) bacteria poses a significant challenge to global public health, contributing to increased morbidity and mortality rates. In this context, the repurposing of existing drugs has emerged as a promising strategy. In this study, hydralazine (HDZ), a vasodilator used as an antihypertensive since 1952, demonstrated antibacterial and antibiofilm activity against both Gram-positive and Gram-negative bacteria. **Methods:** In this study, the antibacterial activity of the antihypertensive hydralazine (HDZ) was evaluated against Gram-positive and Gram-negative strains through minimum inhibitory concentration (MIC), minimum bactericidal concentration (MBC), growth curve with MIC and sub-MIC doses, combinatorial effect with gentamicin, scanning electron microscopy (SEM), molecular docking, and antibiofilm activity. **Results:** The MIC and MBC values ranged from 39.5 to 1.250 μg/mL, respectively. A change in the growth kinetics of the strains was observed when exposed to MIC and 1/2 MIC values, with a delay in the phases of up to 12 h. The combinatorial effect with gentamicin demonstrated an additive and indifferent potential when combined with HDZ. **Conclusions:** Furthermore, hydralazine showed antibiofilm activity against the tested strains, including MRSA. Electron microscopy analysis revealed significant changes in bacterial morphology when exposed to the MIC dose of HDZ for 4 h. The overall results of the study indicate hydralazine as a potential agent in the fight against bacterial infections.

## 1. Introduction

The emergence of multidrug-resistant (MDR) Gram-positive or Gram-negative bacteria is one of the greatest challenges to global public health, as it not only increases morbidity and mortality rates in patients affected by bacterial infections but also leads to a reduction in the therapeutic options available for treating these infections [1,2]. This increases hospital stays and hospital costs, which are estimated to reach US$ 100 trillion by 2050 [3]. In addition, these microorganisms have become significantly resistant to several antibiotics, either through improper use or through bacterial clusters involved in an exopolymeric matrix called biofilm [4,5].

Biofilms are complex structures formed by bacterial communities adhered to surfaces, protected by an extracellular matrix composed of polysaccharides, proteins, and extracellular DNA [6]. This matrix provides high resistance to adverse environmental conditions, including the action of antimicrobials and the immune system. The formation of biofilms is particularly worrying in hospital environments, where they can colonize medical devices, such as catheters and prostheses, as well as inanimate surfaces, contributing to persistent and difficult-to-treat infections [7,8]. Therefore, the constant search for the development of new drugs that are effective in combating MDR is necessary, but the discovery and commercialization of new drugs require a long period of time and high costs, often culminating in failure due to their dangers and high toxicity rates, among other issues analyzed [9].

The secondary use of drugs has gained prominence and popularity on the world stage for introducing the alternative use of a pioneering drug for treating pathologies beyond those intended for its primary use, thus seeking new therapeutic targets [10,11,12,13,14]. A repositioned or reused drug plays a vital role in developing new medications since it can reduce costs by millions of dollars, reducing the risk of failures since its safety has already been elucidated in preclinical and clinical trials, including humans [15].

Hydralazine (HDZ), 1-hydralazine monohydrochloride, is a potent vasodilator used as an antihypertensive agent since 1952. Its mechanism of action occurs through direct modulation of vascular smooth muscle. Under normal conditions, calcium accumulates in the vascular smooth muscle cells via the sarcoplasmic reticulum, resulting in membrane depolarization. This process activates phospholipase C, which in turn promotes the production of inositol trisphosphate (IP3). IP3 facilitates the release of stored calcium, which binds to contractile proteins, inducing muscle contraction and, consequently, vasoconstriction. However, hydralazine acts by decreasing the release of IP3, which reduces the amount of available calcium within the cell and inhibits muscle contraction. This effect leads to vasodilation, reduced peripheral resistance, and thus a decrease in blood pressure, exerting its antihypertensive action [16].

The literature reports the use of HDZ in a repositioned way in cancer therapy [17], with anti-aging benefits [18] and cognitive improvement related to behavioral studies [19], in addition to being a potent antioxidant substance, acting in the elimination of free radicals [20,21,22]. Studies in the literature on the antibacterial activity of hydralazine are scarce. Therefore, its exploratory potential has been increasingly investigated [23]. Thus, the main objective of this work is to report for the first time the repositioning of the antihypertensive drug hydralazine as an antibacterial agent in the fight against standard planktonic and biofilm cells.

## 2. Results

### 2.1. Microdilution Test

The MIC and MBC values of HDZ against the strains tested are shown in Table 1, with MIC values ranging between 39.5 and 625 μg/mL for the strains tested. The *S. epidermidis* strain (ATCC 12238) exhibited the highest sensitivity profile, with a MIC of 39.5 μg/mL, followed by the *S. aureus* strains (ATCC 6538 and ATCC 700698) with MICs of 78.1 μg/mL. The other strains showed MIC values ranging from 625 μg/mL to 156.2 μg/mL. Regarding the MBC values, they ranged from 78.1 μg/mL to 1250 μg/mL, with the lowest value observed for the *S. epidermidis* strain (ATCC 12238). Furthermore, the relationship between the MBC and MIC values demonstrated the bactericidal effect of the compound.

### 2.2. HDZ Effect on Growth Curve

**Figure 1:**
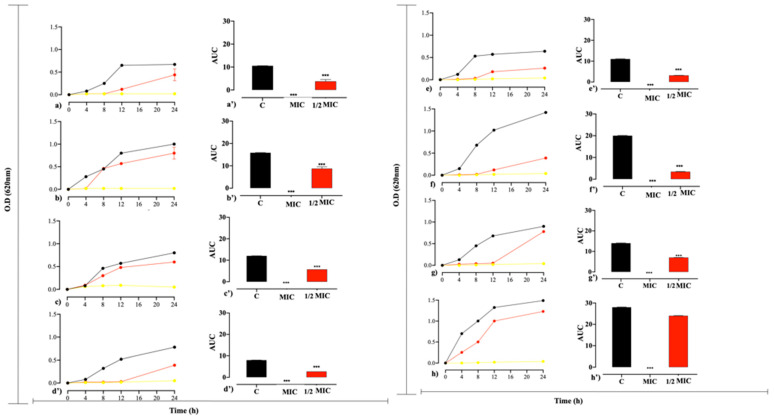
Effect of hydralazine MIC and 1/2 MIC on the bacterial growth curve. (**a**,**a’**) *S. aureus* (ATCC 6538), (**b**,**b’**) *S. aureus* (ATCC 700698), (**c**,**c’**) *S.epidermides*, (**d**,**d’**) *S.mutans* (UA159), (**e**,**e’**) *E. coli* (ATCC 11303), (**f**,**f’**) *K. pneumoniae* (ATCC 700603), (**g**,**g’**) *K. oxytoca* ATCC 13182, and (**h**,**h’**) *P. aeruginosa* ATCC 15442. Bacterial growth was evaluated by area under the curve (AUC), Control (black-line and bar); MIC (yellow-line and bar); ½ MIC (red-line and bar). Significantly different results when *p* < 0.05 compared to the control group (***).

From the growth curve (Figure 1), it was possible to verify that the strains without treatment (control) entered the exponential phase (log) after an approximate period of 4 h of incubation, remaining in growth for more than 24 h, except *S. aureus* and *E. coli* that reached the stationary phase at around 12 h. Furthermore, the strains treated with HDZ at the MIC value showed no bacterial growth, while those treated at the sub-MIC concentration (1/2 MIC) showed a prolongation of the lag phase and a delaying effect on the log for all strains.

### 2.3. Scanning Electron Microscopy

**Figure 2:**
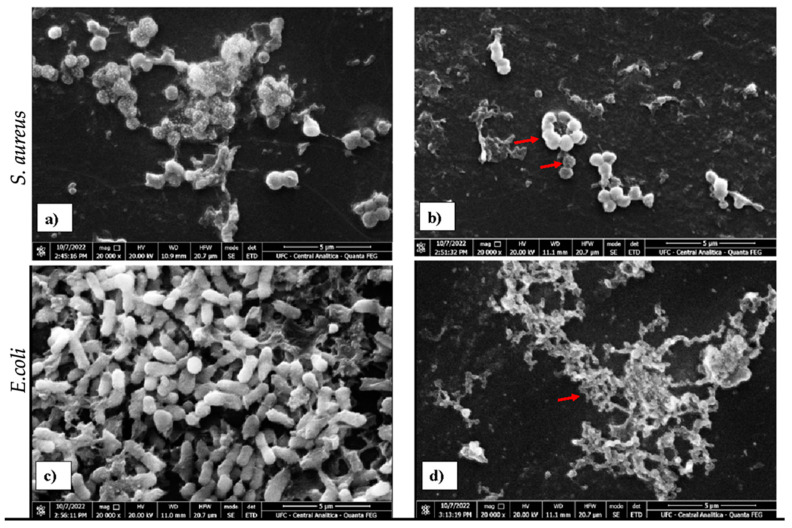
Scanning electron microscopy images of planktonic cells of *S. aureus* (ATCC 6538) and *E. coli* (ATCC 11303), treated with hydralazine (HDZ) at MIC doses. (**a**,**c**) feature untreated groups and (**b**,**d**) feature groups treated with HDZ; red arrows indicate decreased cell density and change in bacterial morphology.

*Staphylococcus aureus* ATCC 6538 and *E. coli* ATCC 11303 and strains treated and untreated with HDZ were analyzed by scanning electron microscopy (SEM). The comparative analysis by SEM showed a reduction in the amount of bacterial cells for a period of 4 h of treatment with HDZ compared to the control group (Figure 2). It is possible to verify a significant change in the morphology and cell density of both strains treated with HDZ in the MIC value, as shown in Figure 2a–d. According to the SEM, morphological alterations were verified in the bacterial strains, and it is possible to verify alterations in the structural arrangement of *S. aureus* (ATCC 6538) and *E. coli* (ATCC 11303) when compared to the control group (Figure 2a,c).

### 2.4. Modulatory Effect of Hydralazine and Gentamicin Combined

To evaluate the modulating activity of HDZ in conjunction with gentamicin, the checkerboard assay was performed, which consists of determining the individual and combined MICs of the antimicrobials, in addition to calculating the values of fractional inhibitory concentration indexes (FICis) for all strains tested, as shown in Table 2.

### 2.5. Antibiofilm Activity

**Figure 3:**
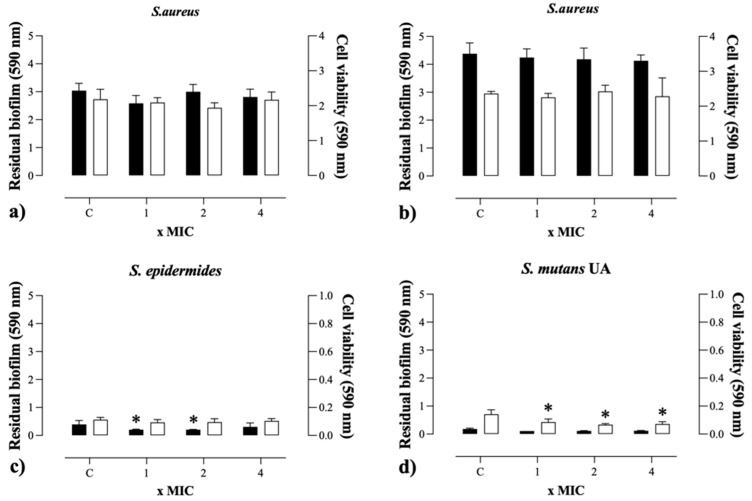

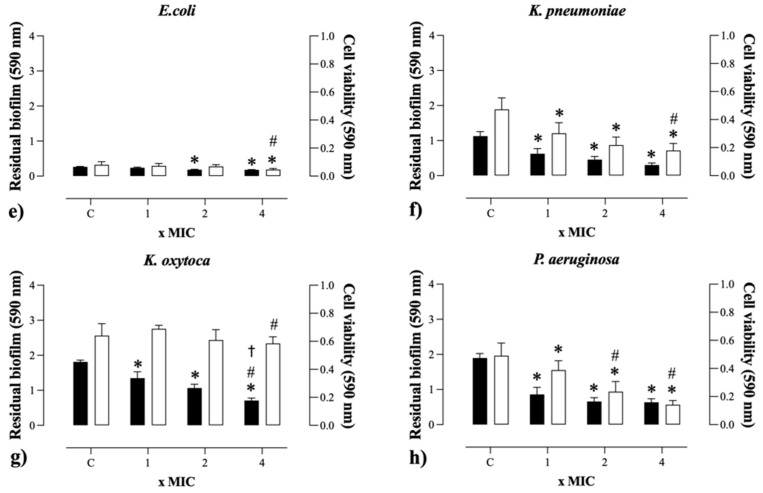
Biomass quantification by crystal violet (CV—black bar) staining and cell viability (MTT—white bar) of increasing concentrations of hydralazine (HDZ), in biofilms of pre-established Gram-positive and Gram-negative bacterial (24 h) after treatment (24 h). (**a**) *S. aureus* (ATCC 6538), (**b**) *S. aureus* (ATCC 700698), (**c**) *S.epidermides*, (**d**) *S.mutans* (UA159), (**e**) *E. coli* (ATCC 11303), (**f**) *K. pneumoniae* (ATCC 700603), (**g**) *K. oxytoca* ATCC 13182, and (**h**) *P. aeruginosa* ATCC 15442. Significantly different results when *p* < 0.05 compared to the control group (*); Difference between the groups treated with CIM an control (†) values at different concentrations and between treatment groups (#).

The effect of HDZ against pre-formed biofilms of Gram-positive and Gram-negative bacteria was evaluated by analyzing residual biomass and cell viability after exposure to different concentrations of the compound, except for *S. aureus*. The results showed significant reductions in biofilm structure and bacterial cell survival following treatments with concentrations equivalent to MIC, 2× MIC, and 4× MIC (Figure 3). These findings indicate that HDZ exhibits antibiofilm potential, affecting both the integrity and viability of cells embedded within the biofilm matrix, which may contribute to the development of new therapeutic strategies for combating persistent infections.

### 2.6. Molecular Docking

**Figure 4:**
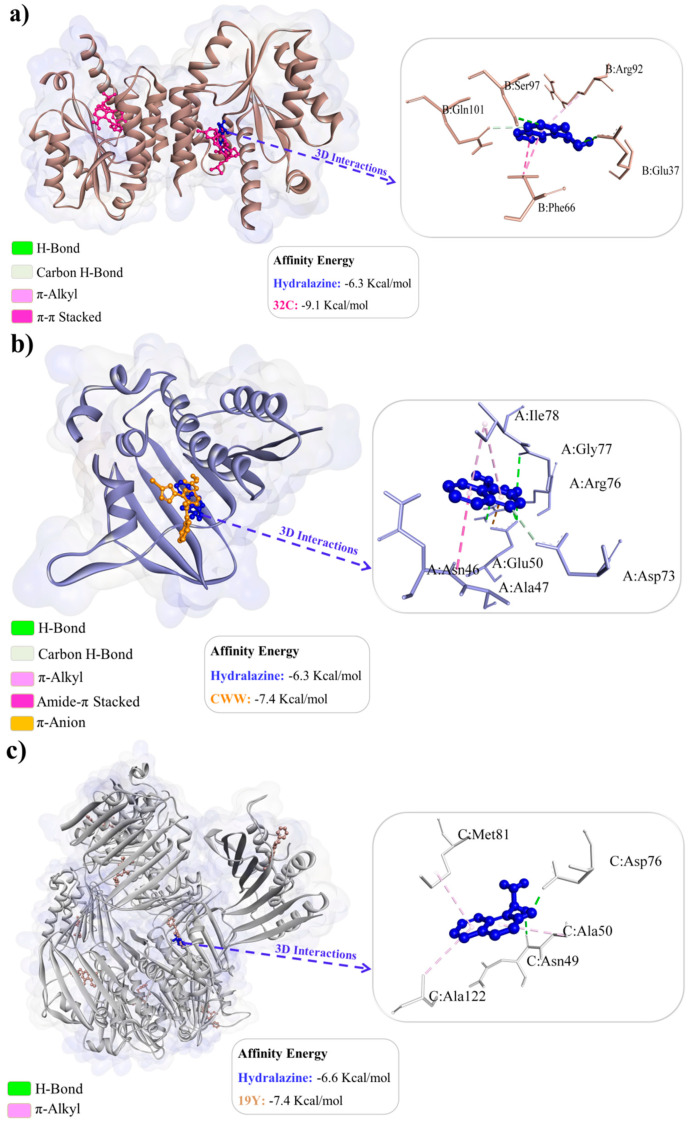
Interaction complex between Hydralazine and the targets Thymidilase kinase (**a**), Gyrase B (**b**), and Topoisomerase IV subunit B (**c**).

The favorable binding affinity energy values confirm the feasibility of complex formation between hydralazine and Thymidylate kinase (−6.3 kcal/mol), hydralazine and Gyrase B (−6.3 kcal/mol), and hydralazine and Topoisomerase IV subunit B (−6.6 kcal/mol) (Figure 4a–c). The redocking of the co-crystallized inhibitor 32C in Thymidylate kinase yielded values of 1.796 Å (RMSD) and −9.1 kcal/mol (binding affinity energy). The binding site consists of the residues Arg 48, Val 51, Leu 52, Phe 66, Ser 97, and Gln 101 [6]. We observed that hydralazine binds to the same region of the binding site as the co-crystallized inhibitor, sharing interactions with residues Phe 66, Ser 97, and Gln 101, indicating that hydralazine exhibits similar activity to 32C.

The redocking of the co-crystallized inhibitor CWW in Gyrase B resulted in values of 1.454 Å (RMSD) and −7.4 kcal/mol (binding affinity energy). The binding site consists of the residues Asn 46, Asp 73, Arg 76, Gly 77, Pro 79, Ile 94, and Arg 136 [7]. We observed that hydralazine binds to the same region of the binding site as the co-crystallized inhibitor, sharing interactions with residues Asn 46, Asp 73, Arg 76, and Gly 77, suggesting a similar mode of action to CWW. The redocking of the co-crystallized inhibitor 19Y in Topoisomerase IV subunit B yielded values of 1.973 Å (RMSD) and −7.4 kcal/mol (binding affinity energy). The inhibitor 19Y interacts with residue Arg 79 [8]. We observed that hydralazine binds to the same region of the binding site as the co-crystallized inhibitor in chain C but does not share common interactions with 19Y.

## 3. Discussion

This study investigated the repurposing of hydralazine (HDZ) as an antimicrobial agent against both Gram-positive and Gram-negative bacteria. HDZ, primarily used in clinical practice for blood pressure management, is available in both oral and injectable forms [23]. However, its potential as a repurposed treatment for bacterial infections has not been fully explored. The antibacterial activity observed in this study was lower compared to the inhibition values typically reported from other repositioned drugs used to combat bacterial infections [24]. Zhang et al. (2022) [25] used niclosamide against different bacterial strains and found MIC values ranging from 500 to 1250 μg/mL for Gram-positive strains. Ref. [26] evaluated the activity of resveratrol against Gram-positive and negative strains and could observe similar values for *E. coli* and *K. pneumoniae* with MIC > 400 μg/mL.

The analysis of the AUC area showed that when comparing the control group with the groups treated with MIC and 1/2 MIC values, there was a significant reduction in bacterial growth for all strains, with Gram-positive strains presenting a higher growth rate after 12 h of exposure compared to Gram-negative strains [12].

The presence of HDZ at MIC and 1/2 MIC concentrations altered the growth kinetics of bacterial strains, as evidenced by plotting growth curves and AUC analysis. Inhibition of bacterial growth was observed in the MIC value during the tested period, while the concentration of 1/2 MIC could delay the log phase, extending the lag phase for up to 12 h. This delay may be due to an inhibition in adhesion according to [27], who, when testing a hydrazide at a concentration of 50 µg/mL, could observe interference with the growth of *Mycobacterium tuberculosis*. Agnihotri et al., also found inhibition of bacterial growth and prolonging of the initial growth phases for Gram-positive and Gram-negative strains at MIC and 1/2 MIC concentrations, respectively, when using silver particles of different sizes in their study, which corroborates to the findings [28].

The evaluation of the time of death curve showed that only the strain of *S. mutans* UA 159 was able to survive when exposed to values of MBC and 2× MBC for a period longer than the time tested due to its resistance profile when tested with different compounds [29]; the other strains died within a period ranging from 1 to 6 h.

HDZ was tested in combination with gentamicin to assess its modulatory activity, significantly reducing the required concentration, with synergistic, additive, and indifferent effects observed among the tested strains. Aminoglycosides are widely used in clinical practice due to their potent bactericidal activity, which inhibits protein synthesis by binding to the ribosomal 30S subunit [30,31,32,33]. In contrast, Kumar et al. (2004) [34] obtained different results in their study with the antihypertensive drug amlodipine combined with aminoglycosides, demonstrating a synergistic potential against Gram-positive and Gram-negative strains.

Similar results were found by [35] when verifying *S. aureus* deformity by SEM in contact with niclosamide at the MIC and 1/2 MIC. Vestergaard and Ingmer [36] also found significant changes in the cell wall of *Pseudomonas aeruginosa* in resveratrol. Thus, HDZ can disrupt the bacterial cell and consequently may have bactericidal activity.

The favorable binding affinity energy values confirm the feasibility of complex formation between hydralazine and Thymidylate kinase (−6.3 kcal/mol), hydralazine and Gyrase B (−6.3 kcal/mol), and hydralazine and Topoisomerase IV subunit B (−6.6 kcal/mol) (Figure 4a–c).

The redocking of the co-crystallized inhibitor 32C in Thymidylate kinase yielded values of 1.796 Å (RMSD) and −9.1 kcal/mol (binding affinity energy). The binding site consists of the residues Arg 48, Val 51, Leu 52, Phe 66, Ser 97, and Gln 101 [37]. We observed that hydralazine binds to the same region of the binding site as the co-crystallized inhibitor, sharing interactions with residues Phe 66, Ser 97, and Gln 101, indicating that hydralazine exhibits similar activity to 32C.

The redocking of the co-crystallized inhibitor CWW in Gyrase B resulted in values of 1.454 Å (RMSD) and −7.4 kcal/mol (binding affinity energy). The binding site consists of the residues Asn 46, Asp 73, Arg 76, Gly 77, Pro 79, Ile 94, and Arg 136 [38]. We observed that hydralazine binds to the same region of the binding site as the co-crystallized inhibitor, sharing interactions with residues Asn 46, Asp 73, Arg 76, and Gly 77, suggesting a similar mode of action to CWW.

The redocking of the co-crystallized inhibitor 19Y in Topoisomerase IV subunit B yielded values of 1.973 Å (RMSD) and −7.4 kcal/mol (binding affinity energy). The inhibitor 19Y interacts with residue Arg 79 [39]. We observed that hydralazine binds to the same region of the binding site as the co-crystallized inhibitor in chain C but does not share common interactions with 19Y.

## 4. Materials and Methods

### 4.1. Antibacterial Solution

Hydralazine (HDZ) was purchased from Sigma-Aldrich (EUA), and the working stock solution was prepared at an initial concentration of 10,000 μg/mL in Tryptone Soy Broth (TSB, Acumedia^®^, Lansing, MI, USA), as well as medium and 1% Dimethylsulfoxide (DMSO), and stored at −20 °C until the commencement of experimental tests.

### 4.2. Microorganisms and Culture Conditions

The *Staphylococcus aureus* ATCC 6538, *Staphylococcus aureus* methicillin-resistant ATCC 700698 (MRSA), *Staphylococcus epidermidis* ATCC 12238, *Streptococcus mutans* UA159, *Escherichia coli* ATCC 11303, *Klebsiella pneumoniae* ATCC 700603, *Klebsiella oxytoca* ATCC 13182, and *Pseudomonas aeruginosa* ATCC 15442 strains were used. The strains were stored at −80 °C in TSB with 20% glycerol. For the cultivation, 50 μL of the stock of strains inoculated in 5 mL of TSB and stored at 37 °C for 18 h were used. The adjustment of the bacterial inoculum was performed at 1 × 10^6^ CFU/mL with an absorbance of 620 nm, using an optical density reader (SpectraMax^®^ Paradigm^®^, Molecular Devices, San Jose, CA, USA).

### 4.3. Antibacterial Activity

The antibacterial activity of HDZ was determined by broth microdilution in 96-well polystyrene plates (KASVI, Curitiba, Brazil) according to the Clinical and Laboratory Standards Institute (CLSI, 2019) protocol [40]. To determine the minimum inhibitory concentration (MIC), the wells were filled with 100 µL of HDZ in concentrations ranging from 19.52 to 2500 μg/mL, and later, with 100 µL of bacterial suspensions adjusted to 1 × 10^6^ CFU/mL. After 24 h of incubation at 37 °C, the lowest concentration capable of inhibiting bacterial growth was considered the MIC. The MBC was determined based on the MIC, with the transfer of 10 μL from the wells where there was no visible microbial growth to plates containing agar TSB. The plates were incubated for 24 h at 37 °C, and MBC was considered the lowest concentration capable of completely inhibiting microbial growth on the agar surface. The experiments were performed in triplicate, with three independent replications.

### 4.4. Kinetic Growth Assay

The growth curve assay was performed for all strains tested in 96-well polystyrene microplates, based on the methodology of [41] with modifications. Bacterial strains were exposed to MIC and 0.5 MIC values for 24 h to verify the interference of HDZ in the bacterial growth kinetics of *S. aureus*, *S. mutans*, *E. coli* and *K. pneumoniae*. MIC plates were mounted as described above with inhibitory and sub-inhibitory concentrations, and MIC and 1/2 MIC results from HDZ were used for analysis and graphing. Readings were performed for 24 h with intervals of 1 h and absorbance at 620 nm in an optical density reader (SpectraMax^®^ Paradigm^®^, Molecular Devices, San Jose, CA, USA). The level of growth was quantified by the area under the curve (AUC), which was calculated as a metric absorbance distribution (620 nm) versus time. The experimental protocol was given by the average evaluation of three replicates.

### 4.5. Scanning Electron Microscopy

Changes in the strains’ morphology were evaluated by scanning electron microscopy (SEM-Inspect S50-FEI Company^®^, Hillsboro, OR, USA). Planktonic cells were cultured in 24-well plates containing 1 × 1 cm glass slides in sterile TSB medium and adjusted to 10^6^ CFU/mL for 24 h at 37 °C. The glass slides were then washed three times with 0.85% saline solution. The cells fixed on the surface of the slides were treated with HDZ at the MIC concentration for 4 h at 37 °C, subsequently washed and dried at room temperature, fixed with 2% glutaraldehyde (Dinâmica, Diadema, Brazil), and then dehydrated with alcohol in concentrations of 10, 30, 50, 70, 90, and 100% for 20 min and dried at room temperature. The slides were placed on carbon tape and aluminum support, coated in gold (Emitech Q150T, Lewes, UK), and visualized via SEM at 20 kW.

### 4.6. Checkerboard Assay

HDZ modulatory activity was evaluated in combination with the antibiotic gentamicin (SANTISA, Bauru, Brazil) according to the methodology of [42]. Initially, HDZ and gentamicin were diluted in TSB broth separately, and later, in a 96-well plate; 50 μL of each compound were added and distributed in different ways. Serial dilution of HDZ was performed on a line and of gentamicin on a column so that each well had a combination of substances at different concentrations. Subsequently, 100 μL of the bacterial inoculum of the tested strains 10^6^ CFU/mL were added, and then the plate was incubated at 37 °C for 24 h. The analysis of the data obtained was calculated, based on the fractional inhibitory concentration index (FICi), as the sum of the MIC of the combined substances, divided by that of the isolated substances and evaluated as synergistic (FICi ≤ 0.5), additive (FICi > 0.5 and ≤ 1), indifferent (FICi > 1 and < 2), or antagonistic (FICI ≥ 2) based on the European Committee for Antimicrobial Susceptibility Testing (EUCAST 2000) [43].

### 4.7. Antibiofilm Activity

The preformed biofilm was treated using MIC-based values (1-, 2-, and 4-fold) of HDZ. Firstly, the mature biofilm was obtained using 200 μL of initial inoculum (~10^6^ CFU/mL) cultured in TSB in 96-well flat-bottom polystyrene plates for 24 h at 37 °C. Then, the wells were washed three times with saline to remove planktonic and loosely attached cells, and the biofilm was treated with 200 μL of antibacterial solutions of HDZ. After 24 h of incubation at 37 °C, residual biofilm biomass was quantified by crystal violet (CV) staining [44], and cell viability was measured by the formation of formazan crystals using Dimethyl-2-Thiazolyl Tetrazolium·Bromide (MTT) [45].

Cell viability was checked by renewing the wells’ culture medium with 100 μL of fresh TSB broth medium and adding 10 μL of 12 mM MTT (Sigma-Aldrich, Darmstadt, Germany) solution to each well. Subsequently, the plates were incubated for 2 h at 37 °C. Next, formazan was solubilized, removing all content from the wells, and 100 μL of DMSO was added to each well and homogenized. Subsequently, the plates were incubated for 10 min at 37 °C and homogenized again. Using a spectrophotometer, absorbance was measured at 590 nm, while 10 μL of MTT stock solution added to 100 μL of culture medium was used as a negative control.

### 4.8. Molecular Docking

The chemical structure of Hydralazine (CID3637) was obtained from the PubChem repository (https://pubchem.ncbi.nlm.nih.gov, accessed on 14 September 2024); the lowest energy confomer was saved at physiological pH using the MarvinSketch code [46] and optimized using the Avogadro code [47], configured to use steepest descent algorithm with 50 interaction cycles, applying the MMFF94 (Merck Molecular Force Field) force field [48,49]. We used the molecular docking technique and methodology proposed by Abo-Salem et al. [50], to investigate the mechanism of action of Hydralazine against the three most important antimicrobial target enzymes [50], where the structures of the Thymidylate kinase targets (PDB ID: 4QGG) [37], Gyrase B (PDB ID: 6F86) [38], and Topoisomerase IV subunit B (PDB ID: 4HZ5) [39] were obtained from the Protein Data Bank repository (https://www.rcsb.org/, accessed on 14 September 2024). In the preparation of the receptors, residues were removed, polar hydrogens added, and Gasteiger charges calculated [51] using the Autodocktools™ code [52].

Molecular docking simulations were performed using the AutodockVina code [53], Lamarkian Genetic Algorithm (LGA), Exhaustiveness 64 [54], and simulation space involving the entire structure of the protein targets across the axes (19.606x, 0.632y, −14.441z), size (110x, 104y, 126z) with thymidylate kinase, axes (64.670x, 28.685y, 53.255z), size (78x, 92y, 104z) with Gyrase B, and axes (18.444x, 37.858y, −3.066z), size (126x, 108y, 118z) with Topoisomerase IV subunit B. Fifty independent simulations were performed, generating 20 poses per simulation and, as part of the best pose selection criterion, the statistical parameter RMSD (Root Mean Square Deviation) was used with values up to 2.0 Å [55] and affinity energy of less than −6.0 kcal/mol [56,57]. To validate the docking simulations, the redocking technique was performed using the inhibitors co-crystallized at the Thymidylase kinase, Gyrase B, and Topoisomerase IV subunit B receptors, being 32C, CWW, and 19Y, respectively. Data analysis was carried out using the UCSF Chimera™ codes [58], Discovery Studio Visualizer, version 16.1.0 (Dassault Systèmes BIOVIA Corp., San Diego, CA, USA), and Pymol [59]. Molecular interactions and hydrogen bonds were calculated using the Discovery Studio Visualizer, version 16.1.0 (Dassault Systèmes BIOVIA Corp., San Diego, CA, USA). Using the values of the distances between donor and recipient atoms, we evaluated the strength of H-bonds, which were considered Strong bonds when they present distances between 2.5–3.1 Å, Average when they present distances between 3.1–3.55 Å, and Weak when they present distances greater than 3.55 Å [60]. 

### 4.9. Statistical Analysis

Assays were performed in triplicate, and results were presented as mean ± standard deviation (SD). Data statistics were evaluated using GraphPad Prism 9.0 software (GraphPad Software, Inc., San Diego, CA, USA), applying analysis of variance (ANOVA) with Bonferroni post-test. Differences between treated and control groups were considered significant when *p* < 0.05, and graphs generated in the growth curve test were used to determine the area under the curve (AUC).

## 5. Conclusions

Hydralazine demonstrated considerable antibacterial potential against various bacterial strains, both Gram-positive and Gram-negative. Molecular docking analyses indicated possible interactions of hydralazine with protein targets essential for bacterial survival, suggesting a mechanism of action directed at key processes in bacterial physiology. Additionally, hydralazine exhibited notable antibiofilm activity, effectively inhibiting biofilm formation. The observation of the morphology of treated bacterial cells using scanning electron microscopy (SEM) suggests, in an exploratory manner, that HDZ may induce structural changes in both Gram-positive and Gram-negative strains. These findings corroborate the results of antibacterial activity evaluations, reinforcing the idea that hydralazine holds promising potential not only to combat planktonic bacterial cells, but also those organized in biofilms. Still, given the pioneering nature of this work, more research is needed to elucidate its action potential.

## Figures and Tables

**Table 1:** Antibacterial activity of hydralazine against Gram-positive and Gram-negative bacterial strains.

Strains Bacterial	Hydralazine		
	MIC (µg/mL)	MBC (µg/mL)	MBC/MIC
*Staphylococcus aureus* ATCC 6538	78.1	156.2	2
*Staphylococcus aureus* ATCC 700698	78.1	156.2	2
*Staphylococcus epidermidis* ATCC 12238	39.5	78.1	2
*Streptococcus mutans* UA 159	625	1250	2
*Escherichia coli* ATCC 11303	625	1250	2
*Klebsiella pneumoniae* ATCC 700603	625	1250	2
*Klebsiella oxytoca* ATCC 13182	156.2	312.5	2
*Pseudomonas aeruginosa* ATCC 15442	156.2	312.5	2

**Table 2:** Fractional inhibitory concentration index (FICi) of hydralazine (HDZ) and gentamicin (GEN) combined against *Staphylococcus aureus* ATCC6538, *S. aureus* (ATCC 700698), *S.epidermides*, *Streptococcus mutans* (UA 159), *Escherichia coli* (ATCC 11303), *Klebsiella pneumoniae* (ATCC 700603), *K. oxytoca* ATCC 13182, and *P. aeruginosa* ATCC 15442.

Strains	Comp.	MIC (µg/mL)		FICi	X-Fold Reduction
		Individual	Combined		
*S. aureus* ATCC 6538	HDZ	78.1	4.87	0.58 (Add)	16
	GEN	625	312.5		2
*S.aureus* ATCC 700698	HDZ	78.1	9.76	0.18 (Syn)	8
	GEN	10	625		16
*S. epidermidis* ATCC 12238	HDZ	39.5	2.45	0.56 (Add)	16
	GEN	625	312.5		2
*S. mutans* UA 159	HDZ	625	156	0.74 (Add)	5
	GEN	10	5		2
*E. coli* ATCC11303	HDZ	625	625	1.13 (Ind)	1
	GEN	10	1.25		8
*K. pneumoniae* ATCC 700603	HDZ	625	312.5	0.56 (Add)	2
	GEN	20	1.25		16
*K. oxytoca* ATCC 13182	HDZ	156.2	78.1	0.56 (Add)	2
	GEN	5	312.5		16

Add: Additive; Syn: Synergistic; Ind: Indifferent.

## Data Availability

The original contributions presented in this study are included in the article. Further inquiries can be directed to the corresponding author.
